# Injuries to the Eye

**Published:** 1907-11-30

**Authors:** 


### Ophthalmology.

### INJURIES TO THE EYE.

### II. PENETRATING WOUNDS DUE TO SHARP INSTRUMENTS.

Attention may now be directed to the second class of injuries—namely, those due to penetrating wounds caused by sharp instruments. Every punctured wound is serious, because it must be looked upon *a priori* as infected, and the retention of septic material within the eye is extremely dangerous. The wound must be carefully freed from all foreign matter, and treated antiseptically.

Should the iris have prolapsed, an attempt should be made to replace it; this is only possible if the case is seen within the first few hours, as the iris soon becomes firmly attached to the cornea and cannot be shifted. If the wound is an incised one, the iris, having once been replaced, will not again prolapse; but if the corneal wound is V-shaped or jagged, the iris will be liable to protrude again. In such a case it would be best to seize the iris with iridectomy forceps, and, withdrawing it gently, cut off the whole of the prolapse. If the case is not seen until too late, and the iris is already firmly attached to the cornea, it should be allowed to heal in this position, and dealt with some weeks later, after the wound has healed and the inflammation subsided.

The eye may be still more deeply penetrated and the lens damaged, in which case a traumatic cataract will form, and the prognosis as regards vision will be in consequence much worse. Should the lens be already opaque and swollen, it may be removed through the wound, but if not, it may be left *in situ* and removed later after the immediate effects of the injury have subsided.

In cases where the vitreous is exposed and appears in the wound, the greatest care must be taken to prevent septic infection; in many cases the prognosis is improved by the escape of the vitreous through the wound; the flow being from within the eye outwards renders it more likely that any septic material will be washed away. If now the conjunctival sac is thoroughly cleansed and kept clean the wound may heal satisfactorily; on the other hand any septic material allowed to infect the vitreous and kept within the eye is bound to result in destruction of the eye.

In many instances a wound penetrating into the vitreous chamber will also involve damage to the retina; should the retina appear in the wound it is wisest to remove the eye, as the possibility of retaining a useful organ of sight is remote and the risk of sympathetic inflammation in the other eye is great.

### III. BLOWS AND CONTUSIONS.

The third class consists of injuries due to blows upon the eye which do not cause an open wound; they may be caused by the fist, balls, stones, or may blunt instrument which usually strikes the eye through the lids; as a result of such injuries the cornea may be driven inwards and the lens backwards.

Since the eye is an elastic capsule the force of a blow of such a nature is transmitted to the opposite pole of the globe, and rupture of the choroid and retina at the back of the eye may result; the immediate effect being that the eye is filled with blood, the extent of the damage cannot be ascertained until this is absorbed. Afterwards it may be found that the pupil remains dilated and fixed. (Traumatic mydriasis.)

In some cases the pupil is altered in shape, due to over-distension or rupture of the fibres forming the sphincter pupillæ. In other cases the iris may be found to have been torn away from its attachment to the ciliary body. (Irido-dialysis.)

At a varying period the lens may turn opaque; this has been caused by rupture of the lens capsule owing to the lens having been forced backwards by the blow. In some cases also the suspensory ligament gives way, the result being a dislocation of the lens.

When the hæmorrhage has cleared sufficiently to make an ophthalmoscopic examination possible, it may be seen that the choroid is torn; if a sufficiently long period has elapsed the position of the rupture is marked by a crescentic white scar, usually partially surrounding the optic disc. A contused blow sometimes causes a rupture of all the tunics of the globe, the site being usually in the anterior section of the eye, at a distance of two to three millimetres from the corneo-sclerotic margin. The cornea, and frequently the conjunctiva, escapes injury. Damage as severe as this does not, however, necessarily involve the loss of the eye, and useful vision may be retained, for the iris and ciliary body being detached from the sclerotic are drawn backwards, and this produces the same effect as if an iridectomy had been performed; when healed the wound can be recognised by its dark colour. The lens also is sometimes forced under the conjunctiva, and can easily be removed at a later date.

It is a cardinal rule in treating injuries to the eye that have produced considerable hæmorrhage and contusion of the globe, to limit active interference to the absolutely essential, as the natural recovery is often far greater than at first may seem probable.

A prolapse of the iris is the condition which needs the most immediate treatment, and if this has not occurred the eye may safely be bandaged and left after the conjunctival sac has been thoroughly washed out.

Sympathetic inflammation is not known to have occurred in less than ten to fourteen days, and usually does not appear for three weeks to a month. So 24 hours' pause before active treatment is often likely to be of more benefit than harm.

